# Pain Trajectories and Predictors Following Uterine Artery Embolization Using a Nitroglycerin–Lidocaine Protocol: A Prospective Study

**DOI:** 10.1002/hsr2.72572

**Published:** 2026-06-08

**Authors:** Fatemeh Mahdavi Sabet, Hamed Ghorani, Ali Mahdavi

**Affiliations:** ^1^ School of Medicine, Tehran Medical Sciences Branch Islamic Azad University Tehran Iran; ^2^ Advanced Diagnostic and Interventional Radiology Research Center (ADIR) Tehran University of Medical Sciences Tehran Iran; ^3^ Department of Radiology, Shariati Hospital Tehran University of Medical Sciences Tehran Iran; ^4^ Department of Radiology, Imam Hossein Hospital Shahid Beheshti University of Medical Sciences Tehran Iran

**Keywords:** intra‐arterial lidocaine, nitroglycerin, post‐embolization pain, uterine artery embolization, uterine fibroids

## Abstract

**Background and Aims:**

Uterine artery embolization (UAE) is an effective minimally invasive treatment for symptomatic fibroids; however, post‐procedural pain remains a major limitation. Intra‐arterial lidocaine administration has shown inconsistent results with concerns regarding vasospasm and incomplete embolization. We addressed this by introducing a sequenced protocol utilizing prophylactic intra‐arterial nitroglycerin. This study aimed to characterize post‐procedural pain trajectories, identify predictors of pain severity, and evaluate the safety of the regimen.

**Methods:**

In this prospective single‐arm study, 34 women with symptomatic uterine fibroids underwent UAE between April and August 2025. To prevent vasospasm, 100 µg of intra‐arterial nitroglycerin was administered into each uterine artery before embolization. Embolization was then performed to near‐stasis, after which 2 mL of 1% lidocaine (20 mg per uterine artery) diluted to 4 mL was infused intra‐arterially. Pain intensity was recorded using the Visual Analog Scale (VAS) at baseline and at 1, 2, 3, 6, 12, and 24 h post‐procedure. Associations between fibroid characteristics and pain were analyzed using correlation analysis.

**Results:**

All procedures were technically successful without complications or vasospasm. The mean patient VAS score peaked at 3 h (6.24 ± 1.67) and gradually declined thereafter. The volume of the largest fibroid correlated positively with mean pain (*ρ* = 0.51 [95% CI: 0.10–0.77], *p* = 0.02) and with pain at 1 and 2 h (both *p* = 0.05). No significant associations were found between pain and uterine size, parity, or adenomyosis. The low‐dose intra‐arterial lidocaine regimen was well tolerated and did not affect embolization outcomes.

**Conclusion:**

The volume of the largest uterine fibroid was associated with greater post‐UAE pain. Prophylactic intra‐arterial nitroglycerin administered before embolization allowed subsequent intra‐arterial lidocaine administration without observed vasospasm. Larger randomized trials are needed to further evaluate and optimize this analgesic approach.

## 1 Introduction

Uterine fibroids are the most common benign uterine tumors, predominantly affecting women in their reproductive years, with an increasing prevalence with age [1, 2]. Uterine artery embolization (UAE), a minimally invasive procedure, was first introduced in 1995 as a successful alternative to traditional surgical treatments for symptomatic uterine fibroids [3]. Since then, it has become a well‐established minimally invasive treatment option, suitable for patients aiming to preserve their uterus [1, 4, 5].

Despite its advantages, a major limitation of the UAE is post‐procedural pain, which is a main feature of postembolization syndrome (PES) [6, 7]. This is believed to be induced by transient ischemia of myometrium and subsequent tissue necrosis [4, 6]. Typically, pelvic pain and cramping emerge within the first 2–3 h after the procedure, intensify over the next 8–12 h, and then gradually subside [8].

Several interventions have been proposed for pain management following UAE, including intra‐arterial lidocaine administration; however, existing literature demonstrates heterogeneity in both methodology and reported efficacy [9, 10, 11, 12]. For instance, Keyoung et al. reported cases of moderate to severe vasospasm when 50 mg of 1% intra‐arterial lidocaine were administered into each uterine artery before embolization [13]. Conversely, Noel‐Lamy et al. reported no adverse events with lidocaine but found a significantly lower rate of complete leiomyoma infarction when lidocaine was mixed with embolization particles compared with administration after the embolization endpoint, with a nonsignificant trend toward the use of fewer PVA particle vials in the lidocaine–PVA group. They hypothesized that even at a diluted concentration of 0.5% lidocaine may induce distal vasospasm, thereby impairing embolization efficacy [11].

The present study aimed to characterize post‑procedural pain trajectories and identify predictors of pain in patients undergoing UAE within the context of a standardized analgesic protocol incorporating post‑embolization intra‑arterial lidocaine. Given that intra‐arterial lidocaine has been associated with vasospasm that may affect embolization efficacy, prophylactic intra‐arterial nitroglycerin was administered to mitigate vasospasm‐related confounding [11, 13]. Nitroglycerin is a nitric oxide–mediated vasodilator widely used in endovascular procedures; it promotes smooth muscle relaxation and arterial dilation, thereby helping maintain luminal patency and facilitating distal embolic particle distribution [14, 15]. Within this standardized framework, we sought to describe the temporal pattern of post‐embolization pain and evaluate its associations with baseline clinical and imaging variables.

## 2 Materials and Methods

We enrolled consecutive women aged 18 years or older with symptomatic, imaging‐confirmed uterine fibroids scheduled for UAE. Exclusion criteria included pregnancy, desire for future fertility, active pelvic infection, known hypersensitivity to lidocaine or iodinated contrast media, severe hepatic or renal dysfunction.

This was a prospective single‐arm observational study approved by the Institutional Review Board (IR.SBMU.RETECH.REC.1404.1100). Consequently, all enrolled participants received the standardized nitroglycerin‐first lidocaine protocol during their UAE procedure. Informed consent was obtained from all individual participants included in the study.

Baseline data, including patient demographics, obstetric history, and clinical symptoms, were prospectively collected. Pre‐procedural imaging (pelvic ultrasound or MRI) was used to document the number of fibroids, dominant fibroid volume, and the largest uterine dimension. Standard pre‐procedure laboratory tests were performed.

All procedures were performed under conscious sedation by a single interventional radiologist (A.M.) with 10 years of experience. Vascular access was via the common femoral or radial artery. To prevent vasospasm, 100 µg of intra‐arterial nitroglycerin was administered into each uterine artery before embolization. Embolization was performed with polyvinyl alcohol (PVA) particles (250–350 µm, escalating to 300–500 or 500–700 µm) to the angiographic endpoint of near stasis, defined as cessation of forward flow for 10 cardiac cycles. Once this endpoint was angiographically confirmed, 2 mL of 1% lidocaine (20 mg per artery), diluted to 4 mL with normal saline, was infused intra‐arterially over 30–60 s. The sequence was repeated on the contralateral side.

Following the procedure, patients received a standardized multimodal analgesia protocol. A continuous intravenous infusion was administered, containing morphine (20 mg) or fentanyl (500 µg) (depending on availability or operator preference), midazolam (5 mg), and ondansetron (6 mg) mixed in 100 mL of normal saline. This was delivered at a fixed rate of 4–8 mL per hour. Because this was a continuous fixed‐rate infusion rather than a patient‐controlled analgesia (PCA) device, individual opioid consumption metrics were not recorded. In addition to the continuous infusion, patients received scheduled intravenous ketorolac (a total of 60 mg) and intravenous acetaminophen (a total of 2000 mg) over the 24‐h monitoring period. Throughout this time, patients were monitored for sedation level, respiratory status, and signs of local anesthetic systemic toxicity (LAST).

The primary outcome was the post‐procedural pain trajectory, measured with a 10‐point Visual Analog Scale (VAS) at baseline (0 h) and at 1, 2, 3, 6, 12, and 24 h; overall mean pain was calculated as the average of these time points. Secondary outcomes included technical success, adverse events, and 1‐month imaging follow‐up.

All data were analyzed using R software (v4.3.2). The normality of continuous variables was assessed using the Shapiro–Wilk test. Normally distributed data were summarized as mean ± standard deviation (SD), whereas non‐normally distributed data were reported as median with interquartile range (IQR). Categorical variables were expressed as frequencies and percentages. Associations between continuous variables, including baseline fibroid volume and mean VAS pain scores, were analyzed using Pearson's or Spearman's correlation coefficients, as appropriate. To account for multiple testing across different time points, *p*‐values for correlations were adjusted using the False Discovery Rate (FDR) method. Correlation estimates are presented alongside their 95% confidence intervals (CIs). A two‐tailed *p*‐value < 0.05 (or FDR‐adjusted *p*‐value, where applicable) was considered statistically significant. For comparisons of group means, differences between means (mean difference) were calculated along with their 95% CIs to provide an estimate of the effect size and its precision.

## 3 Results

Between April and August 2025, 37 patients underwent UAE. Three declined participation, resulting in a final cohort of 34 patients. The median age was 41.0 years (IQR: 6.25; range: 31–48 years). Baseline characteristics are summarized in Table 1.

**Table 1:** Baseline characteristics of the study population (*n* = 34).

Obstetric history	
Prior pregnancy, *n* (%)	12 (35.29%)
Gravidity, mean ± SD^†^	4.75 ± 0.96
Parity, mean ± SD^†^	2.18 ± 0.98
Pregnancy loss/abortion, mean ± SD^†^	2.4 ± 0.55
Clinical presentation, *n* (%)*	
Heavy menstrual bleeding	29 (85.29%)
Back pain	29 (85.29%)
Urinary symptoms	22 (64.71%)
Bloating	20 (58.82%)
Pelvic pressure	15 (44.12%)
Constipation	13 (38.24%)
Dyspareunia	10 (29.41%)
Prior treatments, *n* (%)	
Medical therapy	11 (32.35%)
Prior myomectomy	1 (2.94%)
Fibroid location, *n* (%)**	
Transmural	9 (26.47%)
Intramural	18 (52.94%)
Subserosal	15 (44.12%)
Submucosal	11 (32.35%)
Concurrent imaging findings, *n* (%)	
Adenomyosis	3 (8.82%)

*Note:* Table 1. Baseline demographic, clinical, and imaging characteristics of patients undergoing uterine artery embolization (UAE). Data are presented as numbers (percentages) or means ± standard deviations (SDs), as appropriate. Percentages are based on the total study population (*n* = 34).

* Cumulative percentages exceed 100% as patients frequently presented with overlapping clinical symptoms.

** Cumulative percentages exceed 100% because fibroid locations were assessed on a per‐patient basis, accounting for the presence of multiple fibroid subtypes within a single uterus.

† Calculated only for patients with a history of pregnancy (*n* = 12).

The most common presenting symptoms were heavy menstrual bleeding (85.3%, 29/34) and back pain (85.3%, 29/34). The median number of fibroids was 2 (IQR: 2), and the median fibroid volume was 306.0 cm^3^ (IQR: 235.0; range: 48.0–2000.0 cm^3^). The median largest uterine dimension was 13.0 cm (IQR: 4.5; range: 3.3–19.4 cm). The median baseline hemoglobin was 12.1 g/dL (IQR: 1.35; range: 9.1–14.8 g/dL).

All procedures were technically successful (100%, 34/34). The median number of PVA vials used was 4 (IQR: 2). No major adverse events or LAST were observed.

**Figure 1:**
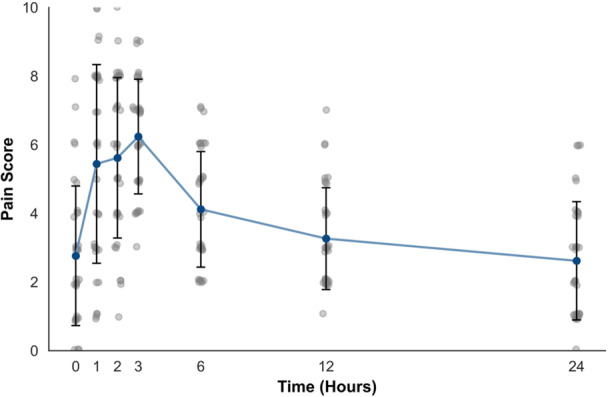
Mean pain scores with standard deviation at different time points following uterine artery embolization (UAE). Pain was measured using a Visual Analog Scale (VAS, 0–10). The solid line indicates the mean pain score, while the error bars represent standard deviation (*n* = 34 patients). Individual data points for each patient are also plotted. Pain peaked at 3 h post‐procedure and gradually declined thereafter.

**Figure 2:**
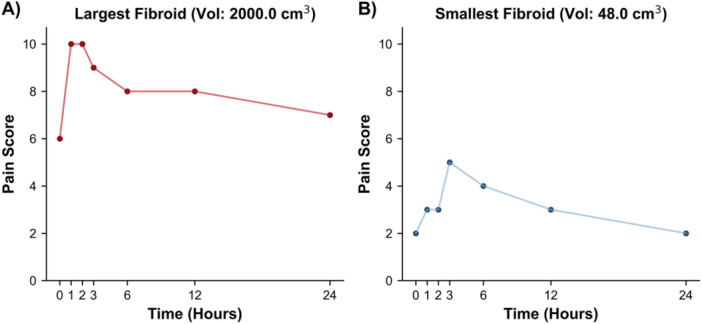
Representative post‐procedural pain trajectories. Pain scores (VAS, 0–10) are shown for two extreme cases. (A) A 39‐year‐old woman with the largest fibroid volume (2000 cm^3^) who required 13 vials of polyvinyl alcohol (PVA) particles. (B) A 37‐year‐old woman with the smallest fibroid volume (48 cm^3^) who was treated via the radial artery using four vials of PVA particles.

Post‐procedural pain varied across time points (Figure 1). Mean VAS scores were 2.77 ± 2.03 at 0 h, 5.38 ± 2.81 at 1 h, 5.59 ± 2.28 at 2 h, 6.24 ± 1.67 at 3 h, 4.12 ± 1.68 at 6 h, 3.27 ± 1.48 at 12 h, and 2.69 ± 1.72 at 24 h. Pain peaked at 3 h post‐procedure and then gradually declined. A significant positive correlation was found between mean pain and fibroid volume (*ρ* = 0.51 [95% CI: 0.10–0.77], *p* = 0.02). As summarized in Table 2, following false discovery rate (FDR) adjustment, fibroid volume was positively correlated with post‐procedural pain at 1 h (*ρ* = 0.53 [95% CI: 0.12–0.78], adjusted *p* = 0.05), and 2 h (*ρ* = 0.54 [95% CI: 0.14–0.79], adjusted *p* = 0.05). Correlations at all other timepoints were non‐significant. Representative pain trajectories for patients at the upper and lower extremes of fibroid volume are illustrated in Figure 2.

**Table 2:** Correlation between baseline fibroid volume and post‐procedural pain scores over 24 h with adjusted and non‐adjusted *p*‐values.

Timepoint	Spearman's *ρ* (95% CI)	Raw p‐value	Adjusted p‐value (FDR)
Baseline (Hour 0)	0.327 (−0.122, 0.665)	0.148	0.205
Hour 1	0.526 (0.122, 0.781)	**0.014**	**0.048**
Hour 2	0.538 (0.138, 0.787)	**0.012**	**0.048**
Hour 3	0.186 (−0.267, 0.572)	0.419	0.419
Hour 6	0.456 (0.031, 0.742)	**0.038**	0.089
Hour 12	0.425 (−0.009, 0.724)	0.055	0.096
Hour 24	0.307 (−0.144, 0.652)	0.176	0.205

*Note:* Bold values represent statistically significant correlations (*p* < 0.05).

Abbreviations: CI, confidence interval; FDR, false discovery rate; *ρ*, Spearman's rank correlation coefficient.

The volume of embolic agent (number of PVA vials) was not associated with pain at baseline (*ρ* = 0.35 [95% CI: −0.01–0.62], adjusted *p* = 0.42), nor at any subsequent time point (adjusted *p* = 0.51–0.89), or with overall mean pain (*ρ* = 0.17 [95% CI: −0.19–0.49], *p* = 0.36). Furthermore, fibroid topography did not appear to influence patient pain, as there were no significant differences in mean pain scores based on the presence or absence of transmural (mean difference = 0.42 [95% CI = −0.90–1.73], *p* = 0.50), intramural (mean difference = −0.73 [95% CI = −1.70–0.23], *p* = 0.13), subserosal (mean difference = −0.23 [95% CI = −1.17–0.71], *p* = 0.62), or submucosal (mean difference = 0.03 [95% CI = −1.07, 1.12], *p* = 0.96) fibroids. Similarly, overall mean pain intensity did not significantly correlate with the largest baseline uterine dimension (*ρ* = −0.19 [95% CI: −0.51–0.18], *p* = 0.31). Additionally, there was a non‐significant negative correlation between the number of fibroids and overall mean pain scores (*ρ* = −0.39 [95%CI: −0.69–0.01], *p* = 0.06); no significant correlations were observed between the number of fibroids and pain scores at any individual time point (adjusted *p*‐values ranging from 0.11 to 0.67).

Stratifying the cohort by baseline clinical characteristics did not change the pain outcomes. Mean pain scores were similar regardless of pregnancy history (mean difference = 0.10 [95% CI = −0.89–1.09], *p* = 0.83), concurrent adenomyosis (mean difference = −0.40 [95% CI = −1.66–0.86], *p* = 0.48), or prior medical treatment for fibroids (mean difference = 0.93 [95% CI = −0.07–1.93], *p* = 0.07).

Ultrasound at 1‐month follow‐up demonstrated no evidence of treatment failure in any of the 34 patients. All patients were discharged within 24 h with continued NSAID therapy. Two patients required conservative management for post‐discharge pain without readmission. No readmissions were recorded during the study period.

## 4 Discussion

In this prospective study, we investigated the predictors of post‐procedural pain following uterine artery embolization (UAE) utilizing a protocol incorporating prophylactic intra‐arterial nitroglycerin and low‐dose lidocaine. Our findings suggest that larger baseline fibroid volume is associated with increased post‐procedural pain. Furthermore, our observations suggest that while the low‐dose lidocaine regimen was safe, it may have been insufficient to blunt the peak of ischemic pain occurring at 3 h post‐procedure. In the absence of a control group, these findings should be interpreted as exploratory and hypothesis‐generating rather than indicative of analgesic efficacy.

A principal finding of our study is the observed positive correlation between baseline fibroid volume and post‐procedural pain. Although a modest positive association between the volume of embolic material and immediate post‐procedural pain was observed, this did not reach statistical significance at any evaluated time point. Post‐UAE pain is primarily driven by ischemia and the subsequent inflammatory response within the uterus, particularly involving the normal myometrium [16]. Prior studies have demonstrated that pain correlates more closely with the degree of myometrial ischemia than with fibroid characteristics alone [6]. In this context, larger or more numerous fibroids often have a more complex and robust blood supply, necessitating a greater embolic load to achieve adequate devascularization, thereby increasing the extent of ischemic injury within adjacent uterine tissue [17]. An illustrative example consistent with this concept has been described by Leo et al. [18], who reported a patient with a large, highly vascular intramural fibroid requiring a substantial embolic load and experiencing severe post‐procedural pain with prolonged hospitalization of 14 days. These observations are consistent with the hypothesis that fibroid size, vascularity, and the extent of embolization collectively influence post‐embolization symptom severity. Given the limited sample size and exploratory design, these findings should be regarded as hypothesis‐generating and require confirmation in larger studies that also account for embolic load, vascularity, and infarction extent.

While our observed peak timing differs slightly from previous reports, which range from an early 1‐h peak [10], to a delayed 6‐h peak depending on the intervention [12], it highlights a fundamental temporal mismatch. Lidocaine has a relatively short half‐life of approximately 90–120 min [9]; and its analgesic effect is therefore largely confined to the early post‐procedural period. Following embolization, arterial occlusion induces hypoxia and cellular necrosis, triggering a progressive release of inflammatory mediators that profoundly amplify visceral nociception [7]. Because this ischemia‐driven inflammatory response continues to evolve over several hours, the transient sodium channel blockade provided by lidocaine dissipates before the pain reaches its maximum intensity [19]. Consequently, the temporal overlap between peak drug efficacy and peak pain remains suboptimal.

Dose may represent an additional factor. Previous studies demonstrating analgesic benefit have generally used higher total doses of intra‐arterial lidocaine (200 mg) [9, 11], whereas lower or moderate doses have shown less consistent effects [10, 20]. The 20 mg dose used in our protocol may therefore fall below the threshold required for sustained analgesic efficacy, particularly in the setting of evolving ischemic injury. To determine whether higher doses might be required for sustained analgesia, future studies should employ controlled, dose‐ranging designs with standardized administration protocols to better define the optimal lidocaine regimen that maximizes pain control while preserving embolization efficacy.

A potential concern with intra‐arterial lidocaine administration is its possible detrimental effect on embolization efficacy [10]. Previous reports have suggested that intra‐arterial lidocaine administered prior to embolization or during the procedure may alter distal flow or induce vasospasm, potentially affecting embolic distribution and treatment outcomes [11, 13]. This concern is supported by findings from Noel‐Lamy et al., who hypothesized that distal vasospasm occurred when mixing lidocaine with embolic particles. In their study, this approach was associated with a trend toward fewer vials of embolic agent used and a significantly lower rate of complete fibroid infarction on follow‐up MRI. Importantly, they noted no such decrease in infarction rates when lidocaine was administered after embolization [11]. Similarly, Keyoung et al. reported that intra‐arterial administration of lidocaine resulted in pronounced vasospasm, leading to premature termination of the study, despite the use of a modified protocol incorporating a reduced dose of 100 mg [13]. Although evidence remains limited and heterogeneous, administering lidocaine after completion of embolization represents a pragmatic strategy to avoid this potential confounding effect while preserving procedural integrity. Furthermore, we deliberately chose to administer the lidocaine only after the true “near stasis” endpoint had been angiographically confirmed. The physiological rationale for this specific endpoint is that it allows for partial recanalization, thereby sparing the healthy myometrium from profound ischemia and inherently reducing post‐procedural pain [21]. To further optimize catheter flow and reduce the risk of vasospasm, intra‐arterial nitroglycerin was administered prior to embolization at a dose of 100 µg per uterine artery, to proactively prevent vasospasm and preserve arterial patency. Finally, we used a lower total dose of lidocaine than in previous studies to reduce potential pro‐thrombotic or vasoactive effects. This strategy was designed to mitigate the risk of incomplete embolization while providing effective pain control.

Given the temporal limitations of lidocaine, there is increasing interest in the use of longer‐acting intra‐arterial anesthetics that may better align with the delayed peak of ischemic pain following UAE. Bupivacaine offers prolonged analgesia due to its higher hydrophobicity but carries concerns regarding cardiotoxicity with intravascular use [22]. Ropivacaine, a structurally similar agent with lower cardiac and CNS toxicity, has demonstrated effective analgesia for up to 9 h after UAE and has shown promising early results in emerging protocols, although further controlled evaluation of intra‐arterial use is needed [22, 23]. While not yet widely adopted in routine practice, these agents may represent a potential direction for future investigation.

Beyond intra‐arterial pharmacologic strategies, superior hypogastric nerve block (SHNB) is increasingly recognized as an effective modality for post‐UAE analgesia, significantly reducing opioid requirements and facilitating early discharge [4, 24]. However, SHNB was intentionally excluded from the present study. Since our primary objective was to isolate the effects of intra‐arterial lidocaine within a standardized protocol, inclusion of a potent regional block would have substantially confounded pain assessment. Future studies should explore whether combining SHNB with optimized intra‐arterial strategies provides additive or synergistic benefit.

Importantly, our experience demonstrated a favorable safety profile. We observed no intra‐procedural or post‐procedural complications attributable to lidocaine. This is consistent with other investigations of intra‐arterial lidocaine in the UAE. Similar to previous studies, no complications or adverse effects were seen in our cohort [9, 13].

This study has several important limitations. Most importantly, the absence of a control group precludes any causal inference regarding the analgesic efficacy of the nitroglycerin–lidocaine protocol. Additionally, although systemic analgesia was standardized across patients, it may have attenuated observable differences in pain attributable to intra‐arterial interventions. The relatively small sample size further limits statistical power and may result in unstable estimates of association; accordingly, observed correlations should be considered exploratory. The intensive multimodal systemic analgesia protocol likely played a dominant role in pain control, potentially masking any modest effect of intra‐arterial lidocaine. Finally, follow‐up imaging was performed using ultrasound rather than MRI, limiting the ability to accurately assess the extent of fibroid infarction. Therefore, definitive conclusions regarding treatment completeness cannot be drawn.

## 5 Conclusions

In conclusion, baseline fibroid volume may be associated with post‐UAE pain and may reflect the overall ischemic burden of the procedure. The use of prophylactic intra‐arterial nitroglycerin in combination with post‐embolization lidocaine was safe but did not appear to substantially alter the expected pain trajectory, potentially due to dose and temporal limitations. These findings should be considered hypothesis‐generating. Future adequately powered randomized controlled trials are warranted to investigate whether the nitroglycerin‐first approach can facilitate the safe use of higher, more effective doses of lidocaine, and to better define the multifactorial drivers of post‐UAE pain.

## Author Contributions

**Fatemeh Mahdavi Sabet:** writing – original draft, writing – review and editing, visualization, formal analysis. **Hamed Ghorani:** project administration. **Ali Mahdavi:** project administration, supervision, data curation, methodology.

## Funding

The authors have nothing to report.

## Disclosure

The lead author Ali Mahdavi affirms that this manuscript is an honest, accurate, and transparent account of the study being reported; that no important aspects of the study have been omitted; and that any discrepancies from the study as planned (and, if relevant, registered) have been explained.

## Ethics Statement

The authors affirm that this study was conducted in full accordance with the ethical principles approved by the Ethics Committee of Salamat Farda Hospital and the Shahid Beheshti University of Medical Sciences Ethics Committee (Ethical No. IR.SBMU.RETECH.REC.1404.1100). All procedures involving human participants complied with institutional ethical standards and with the Declaration of Helsinki (1964) and its subsequent amendments (most recently revised in 2013). Informed consent was obtained from all individual participants included in the study.

## Conflicts of Interest

The authors declare no conflicts of interest.

## Transparency Statement

The corresponding author (Ali Mahdavi) affirms that this manuscript is an honest, accurate, and transparent account of the study being reported; that no important aspects of the study have been omitted; and that any discrepancies from the study as planned have been explained. All authors have read and approved the final version of the manuscript. Ali Mahdavi had full access to all of the data in this study and takes complete responsibility for the integrity of the data and the accuracy of the data analysis.

## Data Availability

The data that support the findings of this study are available from the corresponding author upon reasonable request.

## References

[1] J. Peng , J. Wang , Q. Shu , Y. Luo , S. Wang , and Z. Liu , “Systematic Review and Meta‐Analysis of Current Evidence in Uterine Artery Embolization vs Myomectomy for Symptomatic Uterine Fibroids,” Scientific Reports 14 (2024): 19252. 39164326 10.1038/s41598-024-69754-0 PMC11336172

[2] E. Bedggood , S. Jie , S. Ghosh , et al., “Evaluating Treatment Options for Symptomatic Uterine Fibroids: A Systematic Review and Meta‐Analysis of Effectiveness, Recovery, and Long‐Term Outcomes (MARIE WP1),” Frontiers in Global Women's Health 6 (2025): 1601341. 10.3389/fgwh.2025.1601341 PMC12350422 40821916

[3] J. H. Ravina , J. J. Merland , N. Ciraru‐Vigneron , et al., “[Arterial Embolization: A New Treatment of Menorrhagia in Uterine Fibroma],” Presse Medicale (Paris, France: 1983) 24 (1995): 1754. 8545421

[4] P. Chan , K. Garcia‐Reyes , J. Cronan , et al., “Managing Postembolization Syndrome‐Related Pain After Uterine Fibroid Embolization,” Seminars in Interventional Radiology 38 (2021): 382–387. 34393350 10.1055/s-0041-1731406 PMC8354709

[5] J. Micić , M. Macura , M. Andjić , et al., “Currently Available Treatment Modalities for Uterine Fibroids,” Medicina 60 (2024): 868. 38929485 10.3390/medicina60060868 PMC11205795

[6] A. Ruuskanen , P. Sipola , M. Hippeläinen , M. Wüstefeld , and H. Manninen , “Pain After Uterine Fibroid Embolisation Is Associated With the Severity of Myometrial Ischaemia on Magnetic Resonance Imaging,” European Radiology 19 (2009): 2977–2985. 19533148 10.1007/s00330-009-1481-8

[7] M. G. Waldron , Y. W. Kassamani , A. T. O'Mahony , et al., “Uterine Artery Embolisation of Fibroids and the Phenomenon of Post‐Embolisation Syndrome: A Systematic Review,” Diagnostics 12 (2022): 2916. 36552922 10.3390/diagnostics12122916 PMC9776929

[8] E. Spencer , P. Stratil , and H. Mizones , “Clinical and Periprocedural Pain Management for Uterine Artery Embolization,” Seminars in Interventional Radiology 30 (2013): 354–363. 24436562 10.1055/s-0033-1359729 PMC3835579

[9] S. Duvnjak and P. E. Andersen , “Intra‐Arterial Lidocaine Administration During Uterine Fibroid Embolization to Reduce the Immediate Postoperative Pain: A Prospective Randomized Study,” CVIR Endovascular 3 (2020): 10. 32037475 10.1186/s42155-020-0099-4 PMC7008106

[10] A. Alqahtani , K. Han , S. Y. Kim , et al., “Efficacy of Intra‐Arterial Lidocaine Administration on Pain and Inflammatory Response After Uterine Artery Embolization for Symptomatic Fibroids,” Acta Radiologica 65 (2024): 302–306. 36600596 10.1177/02841851221146517

[11] M. Noel‐Lamy , K. T. Tan , M. E. Simons , K. W. Sniderman , O. Mironov , and D. K. Rajan , “Intra‐Arterial Lidocaine for Pain Control in Uterine Artery Embolization: A Prospective, Randomized Study,” Journal of Vascular and Interventional Radiology 28 (2017): 16–22. 27884686 10.1016/j.jvir.2016.10.001

[12] T. Katsumori , H. Miura , T. Yoshikawa , S. Seri , Y. Kotera , and A. Asato , “Intra‐Arterial Lidocaine Administration for Anesthesia After Uterine Artery Embolization With Trisacryl Gelatin Microspheres for Leiomyoma,” Journal of Vascular and Interventional Radiology 31 (2020): 114–120. 31784332 10.1016/j.jvir.2019.09.007

[13] J. A. Keyoung , E. B. Levy , A. R. Roth , J. Gomez‐Jorge , T. C. Chang , and J. B. Spies , “Intra‐Arterial Lidocaine for Pain Control After Uterine Artery Embolization for Leiomyomata,” Journal of Vascular and Interventional Radiology 12, no. 9 (2001): 1065–1069. 11535769 10.1016/s1051-0443(07)61592-9

[14] K. W. Li , K. W. Liang , W. Y. Liao , et al., “Nitroglycerin (NTG) Infusion for Intraprocedural Vasospasm in Transarterial Microembolization (TAME): A Case Series,” Life 14 (2024): 1413. 39598211 10.3390/life14111413 PMC11595508

[15] L. Wang , I. Horiuchi , Y. Mikami , et al., “Use of Intra‐Arterial Nitroglycerin During Uterine Artery Embolization for Severe Postpartum Hemorrhage With Uterine Artery Vasospasm,” Taiwanese Journal of Obstetrics and Gynecology 54 (2015): 187–190. 25951726 10.1016/j.tjog.2014.05.006

[16] A. G. Vilos , G. A. Vilos , J. Hollett‐Caines , G. Garvin , R. Kozak , and B. Abu‐Rafea , “Post‐Uterine Artery Embolization Pain and Clinical Outcomes for Symptomatic Myomas Using Gelfoam Pledgets Alone Versus Embospheres Plus Gelfoam Pledgets: A Comparative Pilot Study,” Journal of Obstetrics and Gynaecology Canada 36 (2014): 983–989. 25574675 10.1016/S1701-2163(15)30411-4

[17] C. D. Malone , A. Banerjee , M. T. Alley , S. S. Vasanawala , A. C. Roberts , and A. Hsiao , “Pelvic Blood Flow Predicts Fibroid Volume and Embolic Required for Uterine Fibroid Embolization: A Pilot Study With 4D Flow MR Angiography,” American Journal of Roentgenology 210 (2018): 189–200. 29090998 10.2214/AJR.17.18127

[18] L. Leo , R. Thomasset , A. Massaro , et al., “A 40‐Year‐Old Woman With Inoperable Uterine Fibroids Treated With Combined Uterine Artery Embolization and Relugolix,” American Journal of Case Reports 26 (2025): e946334. 39995239 10.12659/AJCR.946334 PMC11868963

[19] D. E. Becker and K. L. Reed , “Essentials of Local Anesthetic Pharmacology,” Anesthesia Progress 53 (2006): 98–109. 17175824 10.2344/0003-3006(2006)53[98:EOLAP]2.0.CO;2 PMC1693664

[20] S. Zhan , Y. Li , G. Wang , H. Han , and Z. Yang , “Effectiveness of Intra‐Arterial Anesthesia for Uterine Fibroid Embolization Using Dilute Lidocaine,” European Radiology 15 (2005): 1752–1756. 15696287 10.1007/s00330-005-2686-0

[21] J. B. Spies , “Recovery After Uterine Artery Embolization: Understanding and Managing Short‐Term Outcomes,” Journal of Vascular and Interventional Radiology: JVIR 14 (2003): 1219–1222. 14551266

[22] J. Steinman and K. T. Tan , “Local Anaesthetics in Interventional Radiology: A Primer for Radiologists on Applications and Management of Complications,” Clinical Radiology 85 (2025): 106917. 40305878 10.1016/j.crad.2025.106917

[23] E. Ho and K. T. Tan , “Novel Pain Management Strategy for Uterine Fibroid Embolization,” CVIR Endovascular 8 (2025): 8. 39841347 10.1186/s42155-025-00516-3 PMC11754559

[24] J. L. Pisanie , C. W. Commander , and C. T. Burke , “Management of Post‐Procedural Uterine Artery Embolization Pain,” Seminars in Interventional Radiology 38 (2021): 588–594. 34853507 10.1055/s-0041-1739161 PMC8612840

